# Does Curcumin Have a Role in the Interaction between Gut Microbiota and *Schistosoma mansoni* in Mice?

**DOI:** 10.3390/pathogens9090767

**Published:** 2020-09-19

**Authors:** Assmaa Anter, Mohamed Abd El-Ghany, Marwa Abou El Dahab, Noha Mahana

**Affiliations:** 1 Zoology Department, Faculty of Science, Cairo University, Giza 12613, Egypt; assmaa99.aa@gmail.com; 2 Botany and Microbiology Department, Faculty of Science, Cairo University, Giza 12613, Egypt; mabdelghany@sci.cu.edu.eg; 3 Zoology Department, Faculty of Science, Ain Shams University, Cairo 11566, Egypt; m_aboueldahab_78@yahoo.com

**Keywords:** *Schistosoma mansoni*, curcumin, mouse gut microbiota, *Pseudomonas aeruginosa*, immune responses, parasitological parameters

## Abstract

There is strong correlation between changes in abundance of specific bacterial species and several diseases including schistosomiasis. Several studies have described therapeutic effects of curcumin (CUR) which may arise from its regulative effects on intestinal microbiota. Thus, we examined the impact of CUR on the diversity of intestinal microbiota with/without infection by *Schistosoma mansoni* cercariae for 56 days. Enterobacteriaceae was dominating in a naive and *S. mansoni* infected mice group without CUR treatment, the most predominant species was *Escherichia coli* with relative density (R.D%) = 80.66% and the least one was *Pseudomonas sp*. (0.52%). The influence of CUR on murine microbiota composition was examined one week after oral administration of high (40) and low (20 mg/kg b.w.) CUR doses were administered three times, with two day intervals. CUR induced high variation in the Enterobacteriaceae family, characterized by a significant (*p* < 0.001) reduction in *E. coli* and asignificant (*p* < 0.001) increase in *Pseudomonas sp.* in both naïve and *S. mansoni*-infected mice, compared to untreated mice, in a dose-dependent manner. Additionally, our study showed the effects of high CUR doses on *S. mansoni* infection immunological and parasitological parameters. These data support CUR’s ability to promote *Pseudomonas sp.* known to produce schistosomicidal toxins and offset the sequelae of murine schistosomiasis.

## 1. Introduction

A large number of parasitic worms and eggs reside in close interaction with gut capillaries, mucosa, and lumen, among these blood flukes of the genus *Schistosoma* (Digenean flatworms, trematodes) [1]. The major *Schistosoma* causative agents of schistosomiasis include *S. mansoni*, *S. haematobium,* and *S. japonicum*. Owing to morbidity and mortality, human schistosomiasis is the most problematic of the human helminthiases [2]. Schistosomiasis or snail fever, one of the major neglected diseases, causes hundreds of millions of infections in several countries of the Middle East, Sub-Saharan Africa, Latin America, and Asia, threatening the economy worldwide [3,4]. About 393 million people in Sub-Saharan Africa are at risk of infection, from which 54 million are infected [5]. Blood fluke *S. mansoni* cercariae released from freshwater *Biomphalaria* snails penetrate human skin and change into schistosomula, which migrate via the blood and lymphatic system to the lung. Thereafter, juvenile worms travel to the venous system of the liver where they mature. After about four weeks, the adult worms migrate into the mesenteries of the intestine, pair up, and become fertile producing hundreds to thousands of eggs per day upon six weeks [6]. Eggs traverse the intestinal wall into intestinal lumen and exit with the stool.

Numerous eggs are retained within the distal vasculature of the intestine and sinusoids of the liver and drift to various tissues, namely the liver. Vigorous immune granulomatous responses to the egg-derived antigens lead to liver fibrosis, intestinal bleeding, and portal hypertension [7], indicating that eggs are responsible for the pathology of schistosomiasis [8]. The immune responses to worm antigens (during the first weeks of infection) are predominantly of the type 1 [9]. Upon egg laying, immune responses rapidly shift to the type 2 axis, characterized by preponderance of interleukin (IL)-13, IL-5, IL-4 [7,10]. This shift of immune responses may be mediated directly or indirectly by modifications in the composition of the intestinal commensal microbiota [11], and promotes mutual benefit, or leads to the eradication of one partner, and affects egg number and viability [12]. In mice treated with antibiotics simultaneously with infection with *S. mansoni,* the host microbiota was found to contribute to triggeringhelminth-specific immune responses [13].

Microbiota such as prokaryotes (bacteria, archaea), viruses, and unicellular eukaryotes reside in the human body, and have a role in individual health. The most colonized organ is the gastrointestinal tract where the colon contains more than 70% of all the microbes in the human body [14]. The major factors that influence the microbiota number and distribution are genetics, diet, antibiotics and the presence of pathogens [15]. Direct contribution of gut microbiota in disease pathogenesis was elucidated by Carding et al. [16]. The association between bacterial and parasitic infection may influence symptom severity, reduce treatment efficacy, or contribute to other symptomatic presentations. The bacterial microbiota also provide protection from colonization by pathogenic microbes, either directly, by producing secondary metabolites such as toxins, antibiotics, enzymes, etc. or competing for nutrients and space, or indirectly, by promoting intestinal barrier function [17].

Treatment and control of schistosomiasis usually depend on only one drug, praziquantel (PZQ) [18]. However, the existence of resistant strains and negative side effects associated with PZQ make alternate treatment strategies attractive [19,20]. Curcumin (CUR) is a major effective component of *Curcuma longa* Linn. (turmeric) rhizomes. It has several pharmacological activities and therapeutic potential against many diseases [21,22]. Antibacterial, antiparasitic, and antifungal activity of CUR was evaluated in vitro, in animal, and in some human studies [23,24]. Despite the low bioavailability and rapid metabolism of CUR [25,26], it is still used as drug in several studies and this may be explained by the interplay between CUR and gut microbiota [27]. The data obtained on the impact of CUR on gut microbiota is still incomplete, although several animal studies showed its efficacy on gut microbial diversity [28].

The current study aims to investigate the impact of infection with *S. mansoni* and CUR administration on outbred mice gut microbiota, in addition to the impact of simultaneous *S. mansoni* infection and CUR treatment on mice gut microbiota number and diversity, the immunological responses to the worm antigens, parasite worm burden, parasite egg counts, and hatchability. This study might provide a new sight for schistosomiasis novel therapy.

## 2. Results

### 2.1. Impact of S. Mansoni Infection on the Composition of Gut Microbiota

Female healthy Swiss albino CD-1 mice ofsix weeks old, were randomly distributed into two groups of four mice each, *S. mansoni* infected and entirely naïve groups. The mice infection was percutaneous with 100 Egyptian strains of *S. mansoni* cercariae (SBSP/TBRI) per mouse. Eight weeks later, both naïve and infected (eight weeks post infection, pi) mice were examined for the composition of gut microbiota.

**Figure 1:**
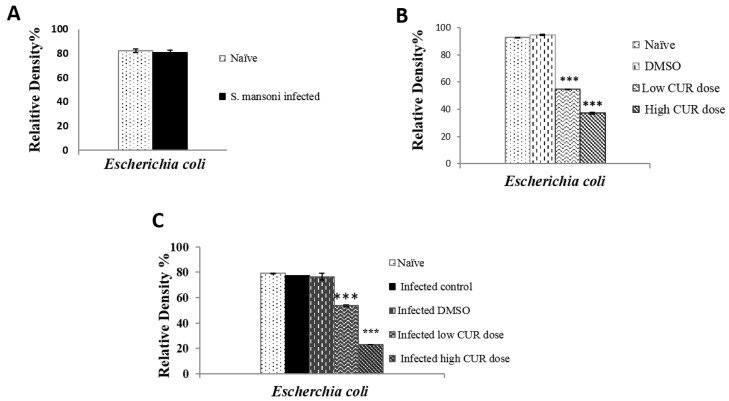
Relative density percentage of *Escherichia coli* isolated from (**A**) naïve and *Schistosoma mansoni* infected; (**B**) naïve, dimethyl sulfoxide (DMSO), low (20 mg/kg b.w.) and high (40 mg/kg b.w.) doses of curcumin (CUR); (**C**) infected, infected DMSO, infected mice groups treated with low and high doses of CUR. Bars are means of four individual mice with (±) standard deviation (SD) around the mean. *** Extremely significant (*p* < 0.001).

**Figure 2:**
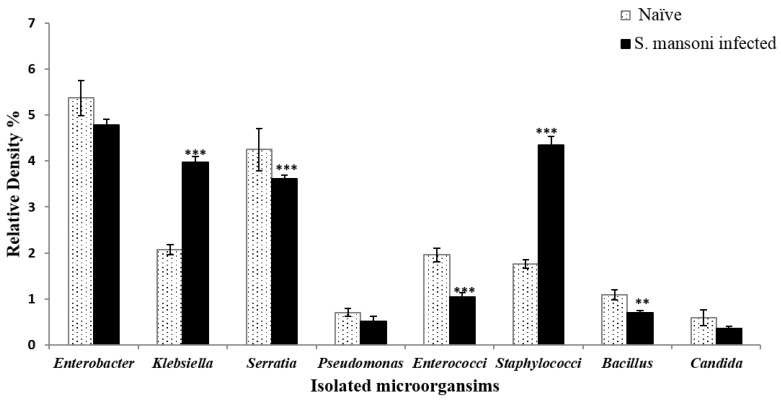
Relative density percentage of microbial isolates, isolated from naïve and *Schistosoma mansoni* infected mice groups. Bars are means of 4 individual mice with (±) standard deviation (SD) around the mean. ** Highly significant (*p* < 0.01), *** Extremely significant (*p* < 0.001).

Mice were dissected and the small intestinal contents (mucus and feces) were collected, diluted, inoculated on a selective, differential medium, and examined by using traditional biochemical methods for identification of recovered microbial species of gut microbiota. In naïve and *S. mansoni* infected mice, Enterobacteriaceae was the most predominant family of Gram-negative bacteria, which can be recovered on a selective and differential medium, represented by the following order *Escherichia coli* (Figure 1), *Enterobacter sp.*, *Serratia sp.*, *Klebsiella sp.*, and *Pseudomonas sp*. (Figure 2). The next family in abundance was Staphylococcaceae including *Staphylococci sp.* followed by *Enterococci sp.,* which belongs to Enterococcaceae family, and Bacillaceae was the least represented family in count exemplified by *Bacillus sp.* (Figure 2). The most dominant species was *E. coli* with R.D% = 80.66% (Figure 1A) and the least one was *Pseudomonas sp*. (0.52%) (Figure 2). By comparison with naïve mice, *S. mansoni* infection induced a significant (*p* < 0.001) increase in R.D% of *Klebsiella sp*. (0.11%) and *Staphylococci sp.* (0.12%) and a decrease in R.D% of the remaining species. On the other hand, insignificant alterations in the growth of *Candida sp.* was observed in *S. mansoni* infected mice compared to naïvemice (Figure 2).

### 2.2. Influence of Curcumin on the Composition of Mice Gut Microbiota

A total of 20 female, six-week-old CD-1 mice were divided into four groups (five mice/each). The first group was left intact, and the others were orally given 1.0 mL/mouse of RPMI medium supplemented with 1% DMSO containing 0 (DMSO controls), 20 (low), and 40 (high) mg/kg b.w. CUR doses. Treatment was given orally three times, with two day intervals. These mice were assayed for the effect of CUR on the intestinal microbiota one week after the last oral administration.

**Figure 3:**
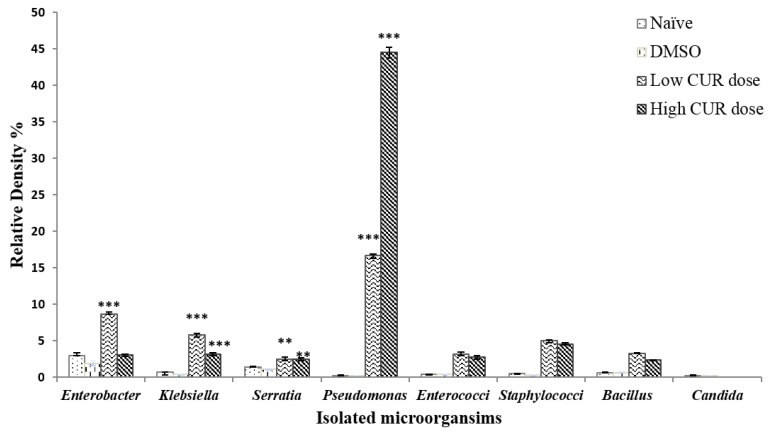
Relative density percentage of microbial isolates, isolated from naïve, dimethyl sulfoxide (DMSO), low (20 mg/kg b.w.), and high (40 mg/kg b.w.) doses of curcumin (CUR) mice groups. Bars are means of five individual mice with (±) standard deviation (SD) around the mean. ** Highly significant (*p* < 0.01), *** Extremely significant (*p* < 0.001).

Administration of DMSO (solvent) did not show any significant differences in the R.D% of microbiota compared to the naïve mice (Figure 3). Mice that received high and low doses of CUR showed a significant difference in microbial diversity of mice gut microbiota. *Pseudomonas sp.* was significantly (*p* < 0.001) the most expressed genus in the Enterobacteriaceae family recovered after high and low CUR doses with R.D% = 62.64% and 14.82%, respectively, when compared with DMSO and naïve control groups that showed R.D% = 0.024% and 0.03%, respectively (Figure 3). Meanwhile, the dominance of *E. coli* was diminished by the administration of the high and low doses of CUR (22.34% and 56.02%, respectively) in comparison with naïve and DMSO controls with R.D% (97.95% and 98.01%, respectively) (Figure 1B). Furthermore, a significant (*p* < 0.000) inhibition of growth of *Candida sp.* in mice administered with low and high CUR doses was detected compared to naïve and DMSO control mice groups (Figure 3). On the other hand, insignificant alterations in Staphylococcaceae, Enterococcaceae, and Bacillaceae families were observed in CUR-administered groups compared to controls (Figure 3).

### 2.3. Schistosomes, CUR, and Gut Microbiota Interactions

#### 2.3.1. Effects on the Microbiota

**Figure 4:**
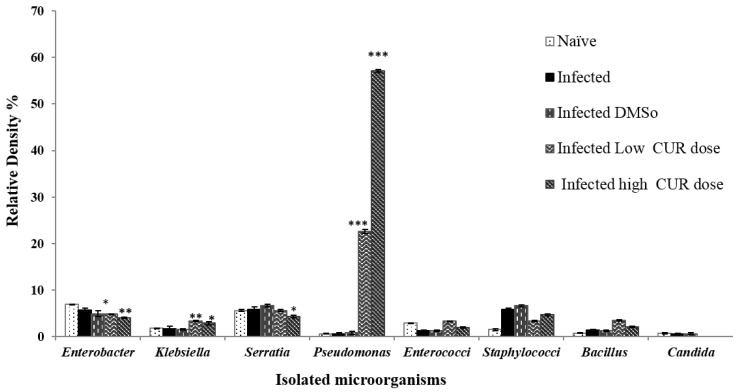
Relative density percentage of microbial isolates, from naïve, infected, infected dimethyl sulfoxide (DMSO), infected mice groups treated with low (20 mg/kg b.w.) and high (40 mg/kg b.w.) doses of CUR. Bars are means of 4–5 individual mice with (±) standard deviation (SD) around the mean. * Significant (*p* < 0.05), ** Highly significant (*p* < 0.01), *** Extremely significant (*p* < 0.001).

It was found that the composition of mice intestinal microbiota infected with *S. mansoni* was not significantly affected by the administration of DMSO (Figure 4). *S. mansoni* infected mice treated with high (40 mg/kg b.w.) and low (20 mg/kg b.w.) doses of CUR induced a great variation in the diversity of genera of intestinal microbiota. *Pseudomonas sp.* (Enterobacteriaceae family) was significantly (*p* < 0.0001) the most dominant species with R.D% = 22.59% and 57.19%, respectively, in the gut microbiota of mice treated with low and high CUR doses in comparison with the R.D% of naïve, untreated, infected, and DMSO infected control groups (0.62%, 0.61%, and 0.81%, respectively) (Figure 4). While *E. coli* counts and R.D% were decreased at a high dose (40 mg/kg b.w.) of CUR treated, infected mice to 22.96%, although it was the most dominant species in the Enterobacteriaceae family of gut-microbiota that appears in naïve, untreated infected, DMSO infected control groups and mice treated with a low dose (20 mg/kg b.w.) of CUR had an R.D% = 79.17%, 77.3%, 76.5%, and 53.58%, respectively (Figure 1C).

Additionally, a significant inhibition of growth of *Candida sp.* and an insignificant alteration in Staphylococcaceae, Enterococcaceae, and Bacillaceae families, was observed in both CUR treated *S. mansoni* infected groups compared to naïve and infected control ones (Figure 4).

#### 2.3.2. Effects on Immune Responses against Schistosome Egg Antigens

**Figure 5:**
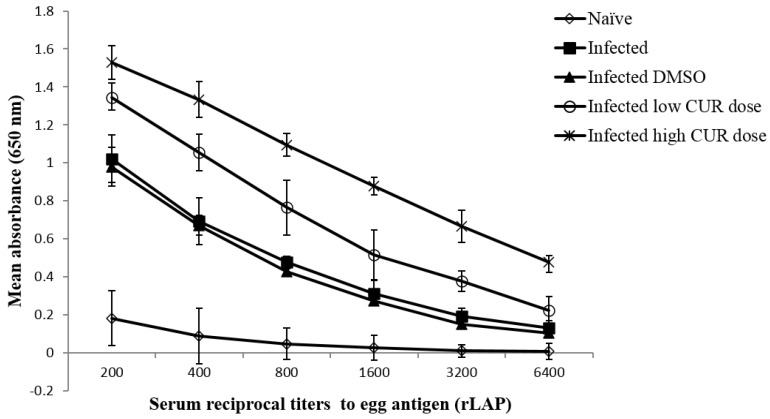
Effect of curcumin (CUR) administration on total serum IgG antibody titers response to recombinant leucine aminopeptidase (rLAP). The data are representative of two independent experiments. Serum of five naïve and *Schistosoma mansoni* infected mice treated with CUR at 0 (DMSO), low (20 mg/kg b.w.), and high (40 mg/kg b.w.) doses were tested for antibody titer to rLAP. Each point represents mean ELISA absorbance (650 nm) of five mice tested in duplicate, and vertical bars denote the standard error (SE) around the mean.

**Antibody Levels.** Naïve and infected mice treated with CUR at 0, 20, or 40 mg/kg b.w. were investigated for serum levels of anti-rLAP IgG antibodies. Compared to naïve mice all *S. mansoni* infected mice displayed significant serum antibody binding to the antigen to a titer of 6400, with mice treated with the high CUR dose constantly showing higher (*p* < 0.01) absorbance values than mice of the other groups (Figure 5). Antibody isotype analysis of sera obtained from individual mice revealed that antibodies binding to rLAP were of IgM and IgG1 isotypes up to a titer of 1:500 and 1:50, respectively, while little (IgG2b and IgA) or no (IgG2a and IgE) other antibody isotypes were detected (Table 1).

**Figure 6:**
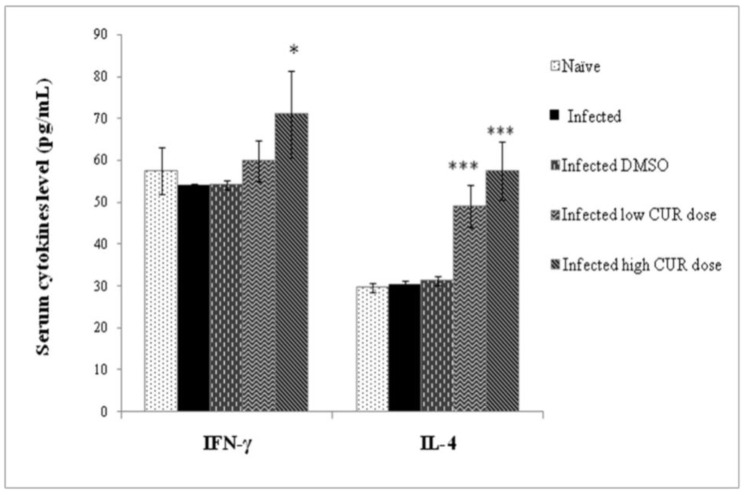
Effect of curcumin (CUR) administration on serum cytokine levels of *Schistosoma mansoni* infected CD-1 mice assessed eight weeks post infection. The data are representative of two independent experiments. Serum offive naïve and *S. mansoni* infected mice treated with CUR at 0 (DMSO), low (20 mg/kg b.w.), and high (40 mg/kg b.w.) doses were tested for circulating cytokine levels. Bars are means of replicate measurements of five individual mice with (±) standard deviation (SD) around the mean. * Significant (*p* < 0.01), *** Extremely significant (*p* < 0.001).

**Cytokine Results.** Eight-week infection with *S. mansoni* failed to significantly alter the levels of circulating IFN-γ or IL-4; treatment of infected mice with low and high CUR doses resulted in a highly significant (*p* < 0.001) increase in circulating IL-4 levels (Figure 6).

#### 2.3.3. Effects on Parasitological Parameters

**Worm Burden.** In the present investigation, treatment of *S. mansoni* infected mice with a high dose of CUR (40 mg/kg B.W) caused a significant (*p* < 0.001) reduction in male, female, and total worm burdens by 65.9%, 61.4%, and 64%, respectively. Otherwise, the changes occurring in DMSO infected control and low CUR dose (20 mg/kg b.w.) treated groups were statistically not significant (*p* ≥ 0.05) compared to the infected untreated mice group, as shown in Table 2.

**Parasite Egg Counts.** Treatment of *S. mansoni* infected mice with high (40 mg/kg) CUR dose caused a significant (*p* < 0.05, 42.1%) reduction in eggs per gram tissue of small intestine but not significantly (*p* ≥ 0.05) in liver egg load. On the other hand, the DMSO infected control group and mice treated with alow (20 mg/kg b.w.) CUR dose did not show any significant reduction in eggs per gram tissue of either the small intestine or liver compared to the infected control group (Table 2).

**Egg Oogram.** The study of the oogram pattern for enumeration of the various egg types is an easy and reliable method of evaluating the therapeutic values of anti-schistosomal drugs. In the current study, treatment with a high CUR dose (40 mg/kg b.w.) elicited a significant increase (*p* < 0.05) in both immature and dead ova with (39.4 and 72.9%, respectively), while a non-significant value (*p* ≥ 0.05) with 13.7% was obtained from mature ones. Likewise, there were no statistically significant (*p* ≥ 0.05) changes in the percentages of dead, immature or mature eggs in the DMSO infected control group and mice treated with a low CUR dose (20 mg/kg b.w.) compared to the infected control group (Table 2).

**Figure 7:**
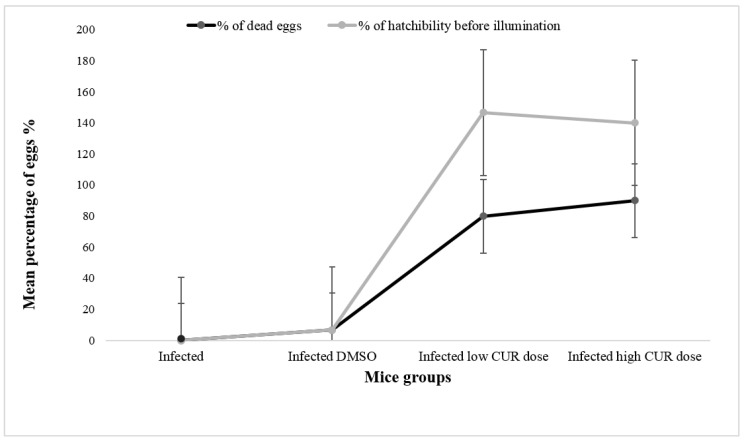
Effect of curcumin (CUR) administration on eggs’ hatchability and viability percentage. The data are representative of two independent experiments. Eggs were obtained from *Schistosoma mansoni* infected mice and infected/treated with CUR at 0 (DMSO), low (20 mg/kg b.w.), and high (40 mg/kg b.w.) doses. The light line shows the percentage of miracidia released before exposure to illumination for 1 h. The dark line shows mean percentage of eggs that failed to hatch (dead). Each point represents mean eggs percentage of five individual miceand vertical bars denote the standard deviation (SD) around the mean.

**Eggs’ Hatchability.** The present study showed thateggs from the small intestine of mice treated with high (40 mg/kg b.w.) and low (20 mg/kg b.w.) CUR doses released a significantly (*p* < 0.0001 and *p* < 0.05, respectively) higher percentage of hatched eggs before exposure to deionized water compared to control and infected DMSO groups (Figure 7). After 1h incubation with deionized water under direct illumination to induce egg hatching, no miracidia were seen in the supernatant of eggs of mice treated with CUR (40 and 20 mg/kg b.w.).

## 3. Discussion

Helminth infection has the ability to alter host metabolic products and immune functions along with modifying bacterial microbiota [29,30]. A loss of microbial diversity in the intestine was found in several human intestinal and extra-intestinal disorders [31]. Zhao et al. [4] suggested that in schistosomes’ infection, egg granulomas in the intestine could influence the differentiation of the gut microbial community under pathophysiological conditions. In the current study, the interrelation between *S. mansoni* infection and the healthy intestinal microbial diversity was investigated and showed that the richness and diversity of intestinal microbiota of CD-1 mice had been changed after *S. mansoni* infection. This result is in agreement with the result of Su et al. [32], who observed significant alterations in gut microbiota of mice infected with helminth. Richness and diversity of intestinal microbiota in mice fecal samples had been changed after *S. japonicum* [4], and *S. mansoni* [11] infections when compared to uninfected controls. Another study on humans showed that *S. haematobium* infection is accompanied by diversity of gut microbiota, despite being bladder infecting schistosomes [33]. This observation is in contrast with Schneeberger et al. [34], who found that few or no modifications in microbiota phyla and diversity in humans infected with *S. mansoni*.

The intestinal content of *S. mansoni* infected mice in the current study was overabundant with the Enterobacteriaceae family (especially *E. coli*) followed by Staphylococcaceae, then Enterococcaceae, while Bacillaceae family was minimal in appearance. Enterobacteriaceae are one of the most abundant bacterial families found in inflammatory gut diseases, including chronic liver diseases [35]. *E. coli* are group of bacteria normally present in the intestinal tract; some are pathogenic, causing vomiting, stomach pain, and diarrhea [36]. On the other hand, gut microbiota in uninfected and *S. japonicum* infected mice were dominated by Firmicutes, Bacteroidetes, and Proteobacteria [34].

A neutral polyphenolic compound (CUR) has numerous pharmacological activities and therapeutic potential against many diseases [37,38]. Therapeutic benefits of CUR may result from either its regulative effect on gut microbiota [28] or its metabolic products released upon the action of gut microbiota [39]. Richness and diversity of microbiota were decreased by orally administered CUR in a non-alcoholic fatty liver disease (NAFLD) rat model [40]. Accordingly, the influence of orally-administeredCUR on the composition of gut microbiota of naive mice was investigated in the current study.

CUR administration led to high variation in the Enterobacteriaceae family including a significant increase in *Pseudomonas sp.* anda significant decrease in *E. coli.* This alteration was concentration-dependent. Similarly, both turmeric extract [41] and CUR [42] were found to significantly decrease the growth of *E. coli*, the most abundant bacteria in the intestinal tract. Oral administration of CUR significantly increased the number of bacterial species in gut microbiota of estrogen deficiency-induced rats, from these *Pseudomonas sp.* and *Serratia* [43]. However, the present study showed insignificant alterations in Staphylococcaceae, Enterococcaceae, and Bacillaceae families of gut-microbiota of CURtreated mice compared to naïve ones. Noticeably, a significant inhibition of *Candida sp.,* one of the most frequent causes of fungal infection, was detected in CUR treated groups compared to control ones. The inhibitory effect of CUR on *Candida sp*. was also detected by Gow et al. [44]; however, this was in vitro. The antifungal CUR effect was explained by its effect on the composition of the lipid membrane that leads to reactive oxygen species (ROS) production causing early apoptosis [45] or its effect on cell wall integrity genes that leads to cell wall damage and membrane permeabilization [46].

The remarkable effects of CUR on isolated gut microbiota may explain the wide range of its beneficial effects. Several studies showed that CUR has positive effects on several diseases by changing the distribution of some gut microbiota [27,28,47]; however, schistosomiasis was not included. For the first time, the present study examined the isolated intestinal microbiota changes in *S. mansoni*-infected mice and their potential impact on treatment outcome based on oral CUR application. Interestingly, oral administration of high and low CUR doses induced alterations on the recovered gut microbiota of *S. mansoni* infected mice, similar to those that occurred in naïve mice, however in a more highly significant manner. This indicates that the dominance of *Pseudomonas sp.* is not linked with disease conditions. We suggest that CUR tends to increase *Pseudomonas sp.*, while schistosomes tend to decrease it, in a dose-dependent manner. This may be explained by Breternitz et al. [48], who found that a cytotoxic protein (MW 28 kDa) from *Pseudomonas aeruginosua* can damage the surface membrane of an adult *S. mansoni* worm, in vitro, by forming membrane pores which are responsible for the breakdown of the permeability barrier. This may be an important mechanism underlying therapeutic benefits of CUR.

Anti-helminth host immunity is dependent on the microbiota [49]. Helminth infections-induced type 2 immune responses have the ability to modify host microbiota that benefit their survival [50,51]. Holzscheiter et al. [13] studied the relation between host’s immune system and *S. mansoni* as well as microbiota; however, the involvement of CUR has never been addressed. The interaction of intestinal helminthes and bacterial microbes dampens or deceives host immunity to expand their survival. Where bacteria tend to suppress Th1/Th17 and helminthes tend to enhance Th2 mechanisms [52], interestingly, both of them tend to activate Treg [53]. Currently, CUR significantly increased IgM and IgG (IgG1 >> IgG2b) antibody production against egg-derived antigen (LAP) in the serum of mice infected with *S. mansoni* cercariae. This is in agreement with Allam [54], who found that CUR treatment augmented IgG and IgG1 responses against soluble egg antigens; however, insignificant changes in serum IgM and IgG2a serum levels of *S. mansoni* infected mice were also detected in his study.

The present study showed that *S. mansoni* infection failed to increase IL-4 (Th2) and IFN-γ (Th1) serum levels while CUR treatment (high dose) significantly (*p* < 0.001, *p* < 0.05, respectively) increased IL-4 and IFN-γ. Accordingly, IL-4 and IFN-γ did not show a significant relationship with schistosome infection status or intensity [51]. However, schistosome eggs’ antigens (the major cause of chronic inflammation in the gut) induced Th2 immune responses (IL-13, IL-5, and IL-4) that suppressed the initial Th1 responses [7], and are responsible for several pathological outcomes [55]. Holzscheiter et al. [13] elucidated the requirement of host intestinal microbiota for schistosomes to initiate Th1 immunopathology. Based on that, *Toxoplasma gondii* required Gram-negative bacteria as *E. coli* in the initiation of intestinal Th1 immunopathology in mice [56]. In agreement with these studies *S. mansoni* infection increased *E. coli* in the gut microbiota of mice and CUR opposed this action. Several bacterial species have been linked with pathogenesis in humans, resulting in either a severe disease burden and/or death [57]. Another study found that CUR inhibited the growth of *E. coli* [41].

In the present study, CUR, especially at high doses, was able to induce protection against schistosomiasis, it significantly reduced worm burdens. Unlike other drugs, it affects adult male and female worms [54]. This may be due to its direct toxic effects such as those reported by Kiuchi et al. [58] and Araújo and Leon [59] on *Toxocaracanis*. Moreover, CUR, in our study, recorded a significant reduction (42.1%) in small intestine egg load; however, it was insignificant in the liver (%) at both low and high concentrations. As in previous studies, the impact of CUR on the microbiota of the intestine, not of the liver, might have a role in this reduction [28,60]. Furthermore, the impact of microbiota on CUR by reduction, demethylation, hydroxylation, and acetylation or the combination of these [61] may release metabolites that affect intestinal egg burdens. The antischistosomal and liver-protective effects of CUR compared with those of PZQ were also studied by [62] and revealed that murine schistosomiasis *mansoni* treated with CUR induced a positive effect by a reduction in worm and egg burdens.

In the current study, CUR treatment showed a significant effect on both immature and dead ova (39.4 and 72.9%, respectively), and, on the contrary, an insignificant effect on mature ova (13.7%) was obtained particularly in high CUR doses. Magalhães et al. [63] and Morais et al. [64] found that the significant decrease in the development of eggs produced by the adult *S. mamsoni* worms did not result from the separation of the coupled adult worms, but it may be related to the in vitro effect of CUR. This was explained by the inhibitory effect of CUR on the ubiquitin–proteasome system, subsequently, reduction in lung-stage schistosomula number, worm burden, and egg count was recorded [65]. Similar to the in vitro study done by [24], CUR was found to increase the hatchability and escape of premature miracidium and led to their death by affecting on the integrity of the shell wall of eggs at low and high concentrations. Pluta et al. [66] showed that the microflora is the way for the fulfillment gaps between poor bioavailability and the enormous effects of CUR on health.

In conclusion, intestinal inflammation due to schistosomiasis affects the intestinal barrier function, increasing bacterial translocation and affecting bacteria number and type. CURis able to offset the sequelae of intestinal barrier dysfunction through alteration in bacterial number, translocation, and inflammation. In addition, an immunomodulatory effect of CUR by increasing IgG and IgG1 antibodies and rising of IL-4 and IFN-γ cytokines level against egg antigen led to a reduction in worm and intestinal egg burdens. Based on this evidence, CUR looks promising in the treatment of schistosomiasis. Additionally, the findings, taken together and with the article by Breternitz et al. [48], suggest a novel mechanism for CUR in vivo schistosomicidal activity.

## 4. Materials and Methods

### 4.1. Curcumin

CUR (1,7-bis (4-hydroxy-3-methoxyphenyl)-1,6-heptadiene-3,5-dione) powder was purchased from Sigma-Aldrich (St. Louis, MO, USA, Catalogue number: 08511). CUR was dissolved in 1% dimethyl sulfoxide (DMSO, Sigma-Aldrich) in RPMI 1640 medium (Lonza, Basel, Switzerland) supplemented with 5% fetal calf serum (FCS).

### 4.2. Animals

Female healthy Swiss albino CD-1 mice, weighing 20 ± 5 g were purchased from the Schistosome Biological Supply Program, Theodore Bilharz Research Institute (SBSP/TBRI), Giza, Egypt and then housed in the animal facility of the Zoology Department, Faculty of Science, Cairo University. The mice were kept under aseptic conditions, fed a standard chow diet, and provided with pure water.

### 4.3. Ethical Consideration

The experiments were approved by the Institutional Animal Care and Use Committee (IACUC) of the Faculty of Science, Cairo University, Egypt, with number CU/I/F/57/19. All the experimental procedures were carried out in accordance with international guidelines for the care and use of laboratory animals performed following the recommendations of the current edition of the Guide for the Care and Use of Laboratory Animals, Institute of Laboratory Animal Resources, National Research Council, USA.

### 4.4. Experimental Design

Experiment 1 was devised to investigate the impact of *S. mansoni* infection on the composition of gut microbiota. Female, 6-week-old CD-1 mice were randomly distributed into two main groups of 4 mice each, *S. mansoni* infected and entirely naïve groups. The mice infection was percutaneous with 100 Egyptian strain of *S. mansoni* cercariae (SBSP/TBRI) per each immediately after shedding from *Biomphalaria alexandrina* as described previously [67]. Examination of microbiota in small and large intestine was performed at eight weeks post infection.

Experiment 2 was conducted to assess the influence of curcumin on the composition of mouse microbiota. A total of 20 female, 6-week-old CD-1 mice were divided into 4 groups (5 mice/each). The first group was left intact, and the others were orally given 1.0 mL/mouse of RPMI medium supplemented with 1% DMSO containing 0 (DMSO controls), 20 (low) and 40 (high) mg/kg b.w. CUR doses. Treatment was given orally three times, with two day intervals. These mice were assayed for the effect of CUR on the intestinal microbiota one week after the last oral injection.

Experiment 3 was performed to examine the interaction of schistosomes, CUR, and microbiota. A total of 20 female, 6-week-old CD-1 mice were percutaneously exposed to 100 *S. mansoni* cercariae, and randomly distributed into 4 equal groups. The first group was left infected and untreated. The remaining *S. mansoni*-infected mice groups were orally administered with 1.0 mL/mouse RPMI medium supplemented with 1% DMSO containing 0 (infected DMSO controls), 20 (infected low), and 40 (infected high) mg/kg b.w. CUR doses starting 6 weeks post infection. In parallel, a group of mice (*n* = 5) was uninfected and untreated and considered as naïve. The mice infection was percutaneous with 100 *S. mansoni* cercariae per each. Treatment was given orally three times, with two day intervals. Mice were assessed for the composition of gut microbiota, serum antibody and cytokine immune responses, and parasitological parameters at one week after the last oral injection, i.e., 8 weeks post *S. mansoni* infection.

### 4.5. Bleeding and Sample Collection

One-week post CUR treatment and about 8 weeks post-infection, blood samples were collected from each mouse (via tail) and sera were separated and kept in aliquots at −20 °C until used (for immunological assay). After that, mice were euthanized with an intraperitoneal injection of 5 mg/kg thiopental sodium (EPICO, 10th of Ramadan City, Egypt) for perfusion and removal of liver and intestine (for microbiological and parasitological assays).

### 4.6. Microbiological Parameters

Small and large intestinal contents (mucus and feces) were collected and diluted (0, 1:10, 1:100, and 1:1000) in deionized sterile water. Then, a calibrated 10 µL of each dilution was inoculated in Nutrient agar (HIMEDIA company REF: M001-500G, LOTNo.0000321511, India) with 5–10% aseptically defibrinated sheep blood and MacConkey agar (OXOID CM0115 No.3-500G, LOT No. 2518594, UK) with Quadrant streaking method [68]. Bacteria were cultured at 37 °C and examined after 24 and 48 h (h) for grown colony count of organisms. Gram stain (EDM, Egypt) was used to differentiate bacteria morphologically, by 40× light microscopy, followed by biochemical reactions [69].

Nine different biochemical reactions were used for identifying the type of organisms. A catalase test was used for differentiating *Staphylococcus sp*. that produced catalase enzyme using 3% hydrogen peroxide (H_2_O_2_) [70]. A coagulase test was used to identify *Staphylococcus aureus,* which produces the coagulase enzyme [71]. A deoxyribonuclease (DNA-ase) test (HIMEDIA company REF: M482-500G, LOTNo.0000408977) was used to identify *Serratia marcescens* which produces DNA-ase enzyme [72]. A Bile Esculin hydrolysis test (OXOID CM0888-500G, LOT No. 1602186) was used as confirmatory test for *Enterococcus sp.* [73]. An oxidase test (Cytochrome oxidase test) (HIMEDIA Company) was used to identify the enzyme cytochrome oxidase which produced from bacteria especially *Pseudomonas sp.* [74]. A Motility Indole Ornithine test (HIMEDIA company REF: M378-500G, LOTNo.0000291389) was used to detect the bacteria which produce indole as *E. coli*, *Citrobacter sp*., and ornithine-decarboxylase activity of enteric *Bacilli* [75]. A Citrate Utilization test (OXOID company CM0155-500G, LOT No. 1740963) was used for detecting the ability of an organism to use citrate as its only source of carbon as in *Citrobacter sp.*, *Klebsiella sp.*, *Serratia sp.*, and *Proteus sp.* [76]. A urease test (OXOID company CM0053-500G, LOT No. 1694661) was used to detect which type has the ability to produce urease enzyme such as *Proteus sp.* and *Klebsiella sp.* [77]. A triple sugar iron (TSI) (OXOID company CM0277-500G, LOT No. 1948263) detects the ability of the fermentation of microorganisms as in *E. coli, Klebsiella sp.* and *Enterobacter sp.* [78].

The developing colonies were counted through colony forming unit/mL (CFU/mL) and the relative density (R.D%) of each microorganism was calculated as percentage of the total microorganisms count.

### 4.7. Immunological Parameters

#### 4.7.1. Antibody Assay

Control and test mice serum antibody levels and isotypes were assessed on an individual mouse basis for binding to egg-derived antigens, namely leucine aminopeptidase in a recombinant form (rLAP, gift of Professor Dr. John P. Dalton, Queen University at Belfast, Northern Ireland) by indirect ELISA as described by Engvall and Perlmann [79] with some modifications.

For the antibody level, wells of polystyrene plates (Costar, Corning, NY, USA) were coated with 250 ng/well rLAP in 100 μL coating buffer (0.1 M Carbonate/bicarbonate buffer, pH 9.6) and incubated overnight at 4 °C. Non-specific sites were blocked with 200 µL/well of blocking buffer (1% bovine serum albumin (BSA, Sigma) in D-PBS) for 1 h. After incubation, all wells were washed 4 times with washing buffer PBS-T (phosphate buffer saline with 0.05% Tween-20). After washing, 100 µL/well of serially dilute serum samples from 1:200 to 1:6400 in diluting buffer (PBS-T and 0.1% BSA) were added in duplicate and incubated at room temperature for 1 hr. Hundred µL/well horseradish peroxidase (HRP)-labeled goat anti-mouse IgG (H + L) conjugate (1:5000 dilution) (KPL, Kirkegaard and Perry Laboratories, Inc., Gaithersburg, MD, USA) was added after washing and incubated for 1 hr. The color developed after adding 100 µL of SureBlue TMB liquid substrate (Sigma) to each well and incubated for 30 min. Reactivity was estimated spectrophotometrically at 650 nm by using an ELISA microplate reader (MultisKan EX, Labsystems, Helsinki, Finland).

For isotyping, mice serum samples were diluted to 1:500 for assessing Ig isotypesIgM, IgG2a, and 1:50 for IgG1, IgG2b, IgA, and IgE levels against rLAP. Biotin-labeled rat monoclonal antibody to mouse IgG1, IgG2b (Pharmingen, San Diego, CA, USA), IgA, and IgE (BioLegend, San Diego, CA, USA), was diluted into 1:500 in washing buffer. Monoclonal antibody to IgM and IgG2a (Pharmingen) labeled with AKP (alkaline phosphatase) was diluted into 1:3000 and 1:1000, respectively. 100 µL/well of each conjugate was added for 1 h at room temperature, and then washed 3 times with 10 mM Tris (10 mM Tris/HCl, pH 7.8 + 150 mMNaCl). Hundred µL of AKP-labeled streptavidin (Promega, Madison, WI, USA) was added to biotin-labeled conjugate wells. While 100 µL/well of paranitrophenyl phosphate liquid substrate (Cal Biochemical, Jolly, Coa) was added to AKP conjugate wells. The plates were covered and incubated for 30 min. at 37 °C. The reactivity was estimated spectrophotometrically at 405 nm.

#### 4.7.2. Cytokines Assay

Level of IFN-γ and IL-4 cytokines was evaluated using sandwich ELISA kits (BioLegend) according to manufacturer’s instruction.

The precoated microwell strips with anti-mouse IFN-γ and IL-4 antibodies were washed with 400 µL/well washing buffer for 2 times before use. All wells were dried and incubated with 100 μL/well of serially diluted standard or sera sample in sample diluents in duplicate. Fifty μL/well of biotin-conjugate (1:100 dilution in assay buffer) was added to all wells, sealed and incubated for 2 h at room temperature. Wells were washed 3 times, and then 100 µL/well Streptavidin–HRP conjugate (1:100 dilution in assay buffer) was added to all wells and incubated for 1 h. After washing 3 times, 100 µL of SureBlue TMB substrate solution was added to each well. The reaction was stopped after 30 min by adding 100 µL/well stop solution. The reactivity was estimated spectrophotometrically at 450 nm. The cytokine concentration was obtained from the standard curve and expressed as pg/mL.

### 4.8. Parasitological Parameters

#### 4.8.1. Worm Burden

Adult *S. mansoni* worms were recovered from on an individual mouse basis by perfusion of the hepatic portal venous system and mesenteric blood vessels 8 weeks after cercarial exposure and 7 days after last CUR treatment as described [80]. The number of males, females, and couples were counted.

#### 4.8.2. Oogram Map

After mice perfusion, small intestinal fragment of about 1 cm was cut longitudinally, rinsed in saline, slightly dried on filter paper, compressed between two glass slides, and examined under a microscope for immature, mature, and dead egg types [81].

#### 4.8.3. Total Egg Counts

Hundred mg pieces of liver and small intestine were evaluated for egg burden following digestion with 4% potassium hydroxide (KOH) digestion for 2 h at 37 °C in water bath with shaking. The number of eggs/g tissue was estimated on an individual mouse basis in pellets obtained after centrifugation and then resuspended in 2% of NaCl in accordance with the total organ weight [80].

#### 4.8.4. Egg Hatchability

*S. mansoni* eggs were retrieved following liver and small intestine tissue digestion in collagenase B (Sigma) according to Dalton et al. [82] with some modifications. Five hundred milligrams liver and small intestine of each CUR untreated or treated *S. mansoni* infected mouse were homogenized and the homogenate then suspended in Hank’s buffer deionized and incubated 3 h with shaking in the presence ofcollagenase B (0.05%, *w/v*). The mixture was sieved, and the filtrate was applied to the top of a Percoll (Pharmacia, Uppsala, Sweden) in 0.25 M sucrose (Sigma). After centrifugation, the pellet (schistosome eggs) was suspended in 1% normal saline for egg count. Then the suspension was centrifuged, and the pellet was added to 5 mL water and exposed to a bright light. The supernatant was removed after 30 min for observing the viability of hatched miracidia then drop of iodine was added for miracidia count.

### 4.9. Statistical Analysis

All data values were tested for normality. The SPSS 20.0 software (SPSS, Chicago, IL, USA) was used in determination of standard deviation (SD) and unpaired Student’s–*t*-2-tailed test was used to analyze the statistical significance of differences between selected values and considered significant at *p* < 0.05.

## Figures and Tables

**Table 1:** Effect of curcumin administration on serum antibody isotypes response to recombinant leucine aminopeptidase (rLAP).

Groups	Mean ± SD					
	IgM	IgG1	IgG2a	IgG2b	IgA	IgE
**Naïve**	0.089 ± 0.010	0.032 ± 0.017	0.066 ± 0.033	0.069 ± 0.008	0.037 ± 0.045	0.004 ± 0.002
**(Cut off)**	(0.109)	(0.066)	(0.132)	(0.085)	(0.127)	(0.008)
**I**	0.284 ± 0.038 *	0.180 ± 0.024 *	0.063 ± 0.018	0.070 ± 0.017	0.045 ± 0.013	0.006 ± 0.005
**ID**	0.219 ± 0.067 *	0.112 ± 0.076 *	0.049 ± 0.036	0.138 ± 0.045 *	0.143 ± 0.019 *	0.007 ± 0.003
**IL**	0.289 ± 0.101 *	0.189 ± 0.056 *	0.041 ± 0.021	0.053 ± 0.034	0.056 ± 0.025	0.007 ± 0.003
**IH**	0.274 ± 0.075 *	0.598 ± 0.130 *	0.036 ± 0.033	0.062 ± 0.020	0.120 ± 0.040	0.007 ± 0.003

The data are representative of two independent experiments whereby sera obtained from five naïve mice and *Schistosoma mansoni* infected untreated (**I**) and treated mice trice with curcumin at 0 (**ID**), low (20 mg/kg b.w.) (**IL**), and high (40 mg/kg b.w.) (**IH**) doses six weeks after infection were tested for antibody isotypes response to rLAP, a major egg antigen. The serum samples were diluted 1:500 (for IgM, IgG2a) and 1:50 (for IgG1, IgG2b, IgA, IgE). * Significant (*p* < 0.05).

**Table 2:** Effect of curcumin administration on parasitological parameters of *S. mansoni* infected mice.

Parameter Counts	I	ID	IL	IH
**Total worm burden**				
Mean ± SD	43.34 ± 8.44	42.0 ± 2.65	42.0 ± 15.25	15.60 ± 10.01
*p* value		0.81	0.9	0.007 ***
Reduction (%)		3.10%	3.10%	64.00%
**Male worm burden**				
Mean ± SD	24.67 ± 3.95	23.0 ± 3.61	26.25 ± 11.15	8.40 ± 5.90
*p* value		0.62	0.83	0.006 ***
Reduction (%)		6.70%		65.90%
**Female worm burden**				
Mean ± SD	18.67 ± 4.46	19.0 ± 4.58	15.75 ± 5.12	7.20 ± 4.87
*p* value		0.93	0.47	0.005 ***
Reduction (%)		77.70%	15.60%	61.40%
**Liver egg counts**				
Mean ± SD	40333 ± 17616	48333.3 ± 14433.76	63750 ± 17100.2	48333 ± 22317
*p* value		0.58	0.14	0.61
Reduction (%)			-	-
**Intestine egg counts**				
Mean ± SD	68500 ± 2121.3	52750 ± 30774.7	53500 ± 33426.5	39666 ± 14982.2
*p* value		0.53	0.58	0.042 ***
Reduction (%)			21.90%	42.10%
**% Immature ova**				
Mean ± SD	33.70 ± 7.42	35.48 ± 12.5	24.9 ± 6.1	20.86 ± 5.22
*p* value		0.84	0.19	0.02 ***
Reduction (%)			27.30%	39.40%
**% Mature ova**				
Mean ± SD	58.38 ± 8.85	57.57 ± 11.03	62.97 ± 5.2	50.25 ± 11.5
*p* value		0.92	0.48	0.32
Reduction (%)		1.70%		13.70%
**% Dead ova**				
Mean ± SD	7.83 ± 4.51	6.96 ± 3.0	12.13 ± 4.6	28.9 ± 10.9
*p* value		0.7	0.22	0.02 ***
Increase (%)			34.90%	72.90%

The data are typical of two independent experiments. *Schistosoma mansoni* infected untreated (**I**) and treated mice with curcumin (CUR) at 0/DMSO (**ID**), low (20 mg/kg b.w.) (**IL**), and high (40 mg/kg b.w.) (**IH**) doses six weeks after infection with 150 ± 50 cercariae and assessed (five per group) for parasitological parameters eight weeks post infection. Differences between CUR-treated and control mice were assessed for significance using Mann–Whitney test. Reduction or Increase % = mean number in untreated control mice—mean number in CUR-treated mice/mean number in untreated control mice × 100. *** Extremely significant (*p* < 0.001).
